# Influence of Non-Newtonian Viscosity on Flow Structures and Wall Deformation in Compliant Serpentine Microchannels: A Numerical Study

**DOI:** 10.3390/mi14091661

**Published:** 2023-08-25

**Authors:** Khemraj Deshmukh, Kunal Mitra, Arindam Bit

**Affiliations:** 1 Department of Biomedical Engineering, National Institute of Technology, Raipur 492010, India; kdeshmukh.phd2019.bme@nitrr.ac.in; 2 Biomedical Engineering, Florida Tech, Melbourne, FL 32901, USA

**Keywords:** serpentine vascular channel, Newtonian and non-Newtonian viscosity, oscillatory shear index, relative residence time (RRT), pressure gradient, axial velocity, shear rate

## Abstract

The viscosity of fluid plays a major role in the flow dynamics of microchannels. Viscous drag and shear forces are the primary tractions for microfluidic fluid flow. Capillary blood vessels with a few microns diameter are impacted by the rheology of blood flowing through their conduits. Hence, regenerated capillaries should be able to withstand such impacts. Consequently, there is a need to understand the flow physics of culture media through the lumen of the substrate as it is one of the vital promoting factors for vasculogenesis under optimal shear conditions at the endothelial lining of the regenerated vessel. Simultaneously, considering the diffusive role of capillaries for ion exchange with the surrounding tissue, capillaries have been found to reorient themselves in serpentine form for modulating the flow conditions while developing sustainable shear stress. In the current study, S-shaped (S1) and delta-shaped (S2) serpentine models of capillaries were considered to evaluate the shear stress distribution and the oscillatory shear index (OSI) and relative residual time (RRT) of the derivatives throughout the channel (due to the phenomena of near-wall stress fluctuation), along with the influence of culture media rheology on wall stress parameters. The non-Newtonian power-law formulation was implemented for defining rheological viscosity of the culture media. The flow actuation of the media was considered to be sinusoidal and physiological, realizing the pulsatile blood flow behavior in the circulatory network. A distinct difference in shear stress distributions was observed in both the serpentine models. The S1 model showed higher change in shear stress in comparison to the S2 model. Furthermore, the non-Newtonian viscosity formulation was found to produce more sustainable shear stress near the serpentine walls compared to the Newtonian formulation fluid, emphasizing the influence of rheology on stress generation. Further, cell viability improved in the bending regions of serpentine channels compared to the long run section of the same channel.

## 1. Introduction

Transfusion of blood in the circulatory network of the human physiological circulatory system addresses multiple metabolic phenomena. Along with the mass transport phenomena, energy transfer is also a crucial feature of circulating blood. Focus on capillary blood transport and rate of fluxes of radial species is significant in comparison to axial mass flux. Further enhancement in the radial transport of chemical species can be achieved by increasing the hydraulic length of capillaries. Radial transport of chemical species is achieved by the natural serpentine arrangement of capillaries [1]. At the same time, the elastic wall of serpentine vascular geometry facilitates vascular regeneration [2]. Hence, a systematic evaluation of the related mechanical properties of blood vessels yields useful markers for analyzing the state of vascular diseases [3,4,5]. However, studies considering the serpentine shape of capillaries and their influence on radial dispersion of species is limited in the literature. Thus, the successful developmental regeneration of vascular capillaries demands a comprehensive mapping of stress distribution on the walls of different serpentine geometry capillaries having micron-dimension radius.

Regenerative materials for vascular capillary play a typical role in sustaining the mechanical properties of vessels while supporting their biological activities. Hydrogel is considered a successful candidate for vascular regenerative devices due to its enhanced transport capabilities, cellular proliferation, and differentiation in its microniche environment [6]. With its superior mixing capabilities [7], it promotes cell functionality over scaffold-based niches [8]. Natural GelMA has inherent stimulating species, which promote cell functionality. Synthetic GelMa lacks mechanical stability and therefore cannot support cell adhesion and attachment [9]. On the other hand, natural biomaterials have the potential to replicate the structure of extracellular matrices to promote cell growth, differentiation, and proliferation [10,11]. Being a gelatin-based bioink, GelMA can intrinsically resolve intricate vascular networks and channels while offering an endothelial cell microniche with the essential properties of native niches.

In addition to media rheology, the transient influx of culture media in the form of physiological flow has also been found to support media transport of chemical species while modulating compliance of the capillary wall [12,13,14,15]. These influx velocity profiles can generate optimized values of shear stress on the inner wall of the capillary vessel structure, thereby supporting cell growth [16,17]. Some studies have evaluated the effect of generic hemodynamic stress on the annular surface of straight and bifurcated tubular channels in the presence of physiological and sinusoidal flow [18,19]. These influx profiles have the capability to develop an adequate amount of stress on the luminal wall containing endothelial cells. Shear stress near the wall of a serpentine model can moderate the viability of cells. The proliferation rate of endothelial cells over a regenerative substrate (hydrogel) is more sensitive to stress distributive patterns [20]. It has been reported that regulated wall shear stress can enhance the growth of endothelial cells and their migration [21]. The interconnected points between the cytoskeleton and the topographical extracellular substrate are also influenced by distributed shear stress. It has been observed that vessel wall compliance of capillaries in the presence of stress enhances the migration properties of seeded cells. Thus, bioprinted serpentine structures with an elastic wall can be an ideal model for enhancing cell viability in the presence of transient influx of culture media. 

The cumulative influence of media material properties (rheological), along with the transient influx, makes the assessment process of shear stress in serpentine models complicated [22,23]. Considering a non-Newtonian rheology of working media, the shear rate is elevated [24] at the bending phase (referring to the sine-wave pattern) of the serpentine structure [25]. This leads to a decrease in the driving pressure [26] and results in elevation of upstream pressure, damaging the smaller arterial blood vessels [27]. Hence, a corrective working pressure of 70 to 100 mmHg can be obtained for implementing pressure boundary conditions for serpentine flow. Blood viscosity increases when the shear rates decrease with increasing vascular diameter or with low flow. 

In the present study, two different serpentine models (S1 and S2) were considered for capillary vessels. A numerical study was conducted to evaluate shear stress mapping on these serpentine models at the physiological boundary condition. Axial velocity pressure and shear rate were evaluated for these serpentine geometries. The material properties of GelMa hydrogels have been previously considered for their structured material properties in order to support cell proliferation [28]. Fluctuation in shear stress with time was evaluated in the form of oscillatory shear index (OSI), which captures the time integral functional form of shear stress near the wall. The OSI quantifies the endothelium’s directional preference to flow-induced shear stress. Hence, temporospatial patterns of the strain rate can be derived from OSI magnitudes. The higher magnitude of OSI at any point of the model contributes to a higher magnitude of executive pressure at that point. The residence period of OSI magnitude can be used to derive the relative residence time (RRT). RRT measures a particle’s residence time on the channel wall. It represents an inverse functionality to that of OSI, i.e., with an increase in OSI value, RRT magnitude decreases, attenuating the promotion of the particle–surface interaction site. Thus, RRT was also evaluated to quantize the regions of lower magnitudes of OSI. 

## 2. Methodology

A two-dimensional CAD model of two different serpentine blood vessels was designed. The influence of fluid on the serpentine wall was realized by a fluid–solid interaction (FSI) module in COMSOL Multiphysics 5.5. Different hemodynamic parameters, namely, axial velocity and shear rate, OSI, RRT, and pressure gradient, were evaluated for two distinct inlet flow boundary conditions. 

### 2.1. Numerical Analysis

**Figure 1:**
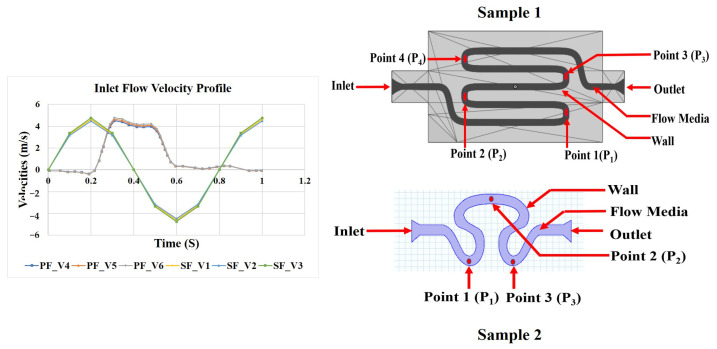
Schematic diagram of the proposed serpentine model of 1 (length is not as per scale; Y1: 5 mm, Y2: 32 mm, Y3: 0.8 mm, Y4: 3 mm, Y5: 5 mm, Y6: 14 mm) and 2 (length is not as per scale; X1: 2.75 mm, X2: 2.75 mm, X3: 2.75 mm, X4: 0.8 mm, X5: 5 mm, X6: 9 mm) with different inlet velocity profiles.

A schematic representation of two different serpentine channels is shown in Figure 1: S-shaped serpentine (model S1) and delta-shaped serpentine (model S2). It also shows the inlet flow boundary conditions, namely, pulsatile and sinusoidal. Mean arterial pressures of 80, 85, and 90 mmHg were considered for this study [29,30]. These pressure values were converted into their corresponding inlet velocities for serpentine geometries 1 and 2. Three different flow rates (corresponding to systolic, diastolic, and mean arterial pressure) based on the transient inlet velocity functions of sinusoidal (SF; V_1_, V_2_, and V_3_) and physiological (PF; V_4_, V_5_, and V_6_) were considered in the study [31]. The physiological functions (waveform) were derived from 4D Laser Doppler data of blood flow across the cross section of the subclavicular artery of a healthy subject. The study was approved by the Institute Ethical Committee (NITRR/IEC/2021/12). The obtained temporospatial functions were interpolated thereafter using the curve-fitting MATLAB 2021a toolbox. Further, the interpolated function with the lowest root mean square error (RMSE) value was selected for the study. The functions corresponding to the inlet flow velocity conditions for V_1_ to V_6_ are given in Table 1. One complete cycle of both sinusoidal and physiological flows is equivalent to the required simulation time, as represented by T [31]. Different study models were formulated for S1 and S2 as tabulated in Table 2.

Fluid flow (with Newtonian and non-Newtonian viscosity formulations for blood equivalent) was evaluated for no-slip boundary conditions near the wall. The following properties of working fluid were used for numerical analysis at 300 K: density = 1066 kg/m^3^ and dynamic viscosity = 3.5 × 10^−3^ Pa·s. The wall of the serpentine channel was modeled using GelMA material properties (density = 1020 kg/m^3^, viscosity = 4.2 × 10^−3^ Pa·s, Young’s modulus = 3.18 KPa) and by considering the Newtonian viscosity formulation. The entire structure was filled with fluid. 

The Navier–Stokes Equations (1) and (2) for non-Newtonian fluids are an extension of the classical equation and include additional parameters to accurately describe the non-Newtonian characteristics of the fluid. The parameters encompass the viscosity coefficient, which is now dependent on the shear rate or stress, and power-law indices, which quantify the fluid’s shear-thinning or shear-thickening characteristics. The viscosity coefficient quantifies the fluid’s opposition to flow, where larger values correspond to greater resistance. Meanwhile, the power-law indices govern the speed at which viscosity alters in response to applied stress. The parameters have physical significance as they are used to quantify the intricate flow characteristics of non-Newtonian fluids. They enable us to create models and gain insights into how these fluids react to external forces and shearing conditions. This is especially critical in other intricate fluids where precise forecasts are vital for optimizing flow patterns and the transport phenomena.

The Navier–Stokes equation was used to represent the momentum of the fluid model. Time-dependent partial differential equation of incompressible Navier–Stokes (Equation (1)) was used for the simulation [31,32]:

(1) $$\rho \frac{𝜕u}{𝜕t}-\eta \nabla^{2}u+\rho \left(u\times \nabla \right)u+\nabla P=0$$

(2) $$\vec{\nabla }\cdot \vec{u}=0$$

where ρ is the fluid density, u is the velocity vector, and η is the dynamic viscosity. The non-Newtonian power-law formulation was also considered for evaluating the viscosity of the culture medium. The power-law viscosity model was represented by Equation (3), where *η* is the viscosity, *n* is the power-law constant, *K* is the flow consistency index, and γ is the shear strain rate (s^−1^). The *n* and *K* were selected as 0.66 [33] and 1, respectively, in the present study [34]:

(3) $$\eta =K\gamma^{n-1}$$

Four different points corresponding to positions P_1_ (on rear region 1), P_2_ (on neck region 1), P_3_ (on rear region 2), and P_4_ (on neck region 2) were identified on sample 1. Similarly, for sample 2, three different points, P_1_, P_2_, and P_3_, were identified on the neck, abdominal, and rear region, respectively (as shown in Figure 1). These regions of samples 1 and 2 are the downstream region immediately after curvilinear orientation of the ascending and descending phases of the serpentine structure, and they are likely to be sensitive to vortex formation within the serpentine model. The pressure, axial velocity, and shear rate over an entire cardiac cycle (T) were evaluated at distinct set points of T/3, T/2, and T cycles.

One of the key factors affecting OSI is wall shear stress (WSS). A high OSI can result when the direction of WSS varies quickly over time as it does in oscillatory flows [35]. The WSS was calculated near the wall of the serpentine vascular channels using Equation (4):

(4) $$\mathrm{WSS}=\frac{1}{T}\int_{0}^{T}\left|\tau_{w}\right|\mathrm{dt}$$

where $\tau_{w}$ = stress magnitude at all 24 and 13 points for samples 1 and 2, respectively. 

**Figure 2:**
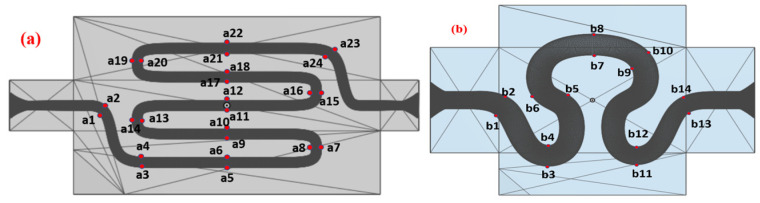
Different points for WSS, OSI, and RRT calculation of (**a**) model S1 and (**b**) model S2.

The WSS was calculated for the no-slip wall boundary condition for both samples 1 and 2 for physiological flow for velocity model V_6_. The typical reason for selecting V_6_ was higher velocity magnitude compared to other velocity models as it leads to a higher velocity gradient near the wall, which in turn leads to maximum variation in the wall shear stress value. The time integral of WSS was calculated at different study points, as indicated in Figure 2. The WSS was measured at all 24 and 14 ROI points for samples 1 and 2, respectively, as the maximum WSS variation was observed at those points.

The WSS plays a critical role in the development of OSI and can be a useful indicator of the hemodynamic forces. Significant variation of the WSS magnitude was used to evaluate the behavior of oscillatory shear index (OSI) [36]. In the present study, OSI was evaluated using Equation (5):

(5) $$OSI=\frac{1}{2}(1-\frac{\left|(\frac{1}{T})\int_{0}^{T}WSSdt\right|}{(\frac{1}{T})\int_{0}^{T}\left|WSS\right|dt})$$

where *T* = total time cycle of cardiac impulse (or simulation duration).

The value of OSI at different study points for serpentine geometries 1 and 2 were computed using Equation (5). 

The magnitude of WSS was influenced by the coaxial variation (curl) of the serpentine model. Unsteady flow behavior within the vascular structure was used to evaluate computational analysis time. The particle spent time [37] within the serpentine channel was estimated by relative residence time (RRT) using Equation (6):

(6) $$\mathrm{RRT}\sim {\left[\left(1-2.0\times OSI\right)\times WSS\right]}^{-1}$$

From Equation (6), it can be observed that the RRT parameter includes the effects of both OSI and WSS [38]. 

Fluid flow and hemodynamic parameters were solved by COMSOL Multiphysics. Laminar flow and solid mechanics modules were used to evaluate these parameters. Fluid-Structure Interaction Multiphysics was used to establish the relationship between two physics. Fine element size was used for meshing the entire geometry with 0.2293 minimum element quality. The fluid–solid contact equation was solved using the Multifrontal Massively Parallel Solver (MUMPS) in COMSOL Multiphysics 5.5 [39]. The linearity of a system with real nonsymmetrical and symmetrical positive attributes can be analyzed using MUMPS [40]. MUMPS uses Gaussian elimination to resolve a linear equation based on sparse data used in a distributed memory system [41]. The following methods and termination parameters were used—nonlinear method: constant (Newton), termination technique: iteration or tolerance, and termination criteria: solution and residual. Sensitivity and uncertainty analyses were performed to observe the variability of the output parameters for different input parameters following previously used methods [31]. 

### 2.2. Grid Convergence Index

A grid convergence test was performed in order to establish the ideal grid size for the study, ensuring that the results were not influenced by the grid resolution. The grid convergence index (GCI) is a quantitative metric used to assess the level of convergence in a grid refinement study [42]. Both the serpentine models S1 and S2 were discretized using three distinct element sizes. This discretization was carried out for a sinusoidal flow with velocity V3. The pressure at point P_1_ was assessed for a duration of one complete cycle considering various sizes of elements. The order of convergence was evaluated using Equation (7).

(7) $$p=ln\frac{\left(f_{3}-f_{2}\right)}{\left(f_{2}-f_{1}\right)}/\ln\left(r\right)$$

The variables *f*_1_, *f*_2_, and *f*_3_ represent the parameter values of pressure at P_1_ for three distinct grid sizes. Based on the analysis of Equation (7), the variable *p* was determined and the refinement ratio ‘*r*’ was computed based on the grid spacing. Upon performing necessary calculations for the relevant parameters, the grid convergence index (GCI) was determined. The GCI was calculated using the Richardson extrapolation method, which relies on the estimation of the fractional error [42]. The GCI was calculated using Equation (8), and the results are listed in Table 3.

(8) $$GCI=\frac{F_{s}\left|e\right|}{\left(r^{p}-1\right)}\times 100\%$$

## 3. Results

**Figure 3:**
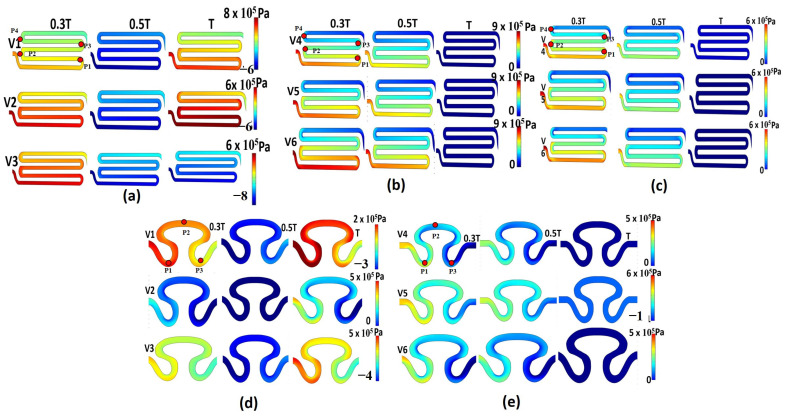
Contour plot of pressure for different models: (**a**) model S1, non-Newtonian viscosity with sinusoidal flow; (**b**) model S1, non-Newtonian viscosity with physiological flow; (**c**) model S1, Newtonian viscosity with sinusoidal flow; (**d**) model S2, non-Newtonian viscosity with sinusoidal flow; (**e**) model S2, non-Newtonian viscosity with physiological flow.

Hemodynamic parameters, including pressure, axial velocity, and shear rate, are important regulators that can impact cell growth. All S-shaped serpentine model analyses for the S1 model were grouped as I, II, and III, while the delta-shaped serpentine model analyses for the S2 model was grouped as conditions IV and V. No Newtonian study was added for the S2 model because the Newtonian model had shown significantly lower spatial variation for strain rate in comparison to non-Newtonian models in the S1 model. Hence, this model was not considered further in the study. Figure 3 represents the contour plot of pressure for all groups. For the non-Newtonian viscosity formulation with sinusoidal flow (model I, Table 2), maximum pressure of $8\times 10^{5}$ Pa and minimum pressure of $-6\times 10^{5}\;Pa$ were developed for inlet flow velocity condition V_1_ on its rear region 1 (P_1_) and neck region 2 (P_2_), respectively (refer to Figure 3a). It was observed that for all inlet velocity conditions in the S1 model, the pressure decreased as fluid flowed from the rear region 1 (P_1_) to the neck region 2 (P_4_) by the end of the complete cycle. This can be attributed to the development of localized Coriolis vortex formation due to the occurrence of abrupt angle of inclination on the downstream of the flow path. The contour plot of pressure for the non-Newtonian viscosity formulation with physiological flow (model II) is shown in Figure 3b. It was observed that in due course of time, the localized pressure value decreased with respect to space directed from the inlet to the outlet for inlet flow velocity conditions V_4_ and V_5_. For the Newtonian viscosity formulation with physiological flow (model III, Table 2), the magnitude of pressure was constant for all flow velocity conditions at the end of the study cycle. Maximum pressure of $6\times 10^{5}$ Pa was observed for all flow velocity conditions for model III (physiological flow with Newtonian viscosity of serpentine model 1, see Table 2). At the edge of neck region 1 (P_2_) of serpentine model S1 at the T/3 cycle, maximum pressures were identified, as shown in Figure 3c. The pressure contour of non-Newtonian viscosity formulation for sinusoidal flow of serpentine model S2 (model IV, refer Table 2) is shown in Figure 3d. It was observed that the maximum pressure gradient was obtained in the neck region P_1_ for inlet flow velocity conditions V_2_ and V_3_. Similarly, for inlet flow velocity condition V_1_, maximum pressure gradient of $2\times 10^{5}$ Pa was recorded in between the neck region (P_1_) and abdominal region (P_2_) of sample S2 at the end of the T cycle. For model V (physiological flow with non-Newtonian viscosity of serpentine model S2, refer Table 2), the contour plot showed pressure values ranging from $-1\times 10^{5\;}\;\mathrm{to}\;6\times 10^{5\;}$ Pa for inlet flow velocity condition V_5_ in the neck region P_1,_ as shown in Figure 3e. For the other two flow velocities V_4_ and V_6_, the maximum pressure gradient was observed near the neck region P_1_. Overall, the results showed that for all flow boundary conditions, Newtonian fluid had a small pressure gradient, so it could easily flow through the channel. However, the pressure drop considering the non-Newtonian fluid flow formulation was substantially larger (Figure 3a,c). In contrast, the curvature of the fluid interface was maximum in the serpentine model S1, which produced a greater maximum pressure gradient compared to serpentine model S2, as shown in Figure 3a,d.

**Figure 4:**
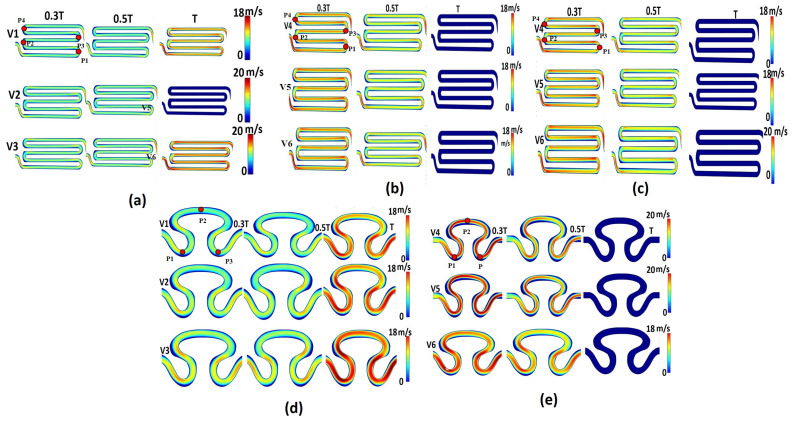
Contour plot of axial velocity for different models: (**a**) model S1, non-Newtonian viscosity with sinusoidal flow; (**b**) model S1, non-Newtonian viscosity with physiological flow; (**c**) model S1, Newtonian viscosity with sinusoidal flow; (**d**) model S2, non-Newtonian viscosity with sinusoidal flow; (**e**) model S2, non-Newtonian viscosity with physiological flow.

The flow pattern in a serpentine channel is not uniform, with areas of higher and lower pressure along its length. The channel geometry can influence the flow pattern, generating eddies or vortices, and change the axial velocity of fluid. The axial velocity contour profiles for all five models are shown in Figure 4. The magnitude of axial velocity for the non-Newtonian viscosity formulation with sinusoidal flow of sample S1 (model I, Table 2) was found to be maximum (20 mm/min) on the rear region (P_1_ and P_2_) and neck region (P_1_ and P_2_) for inlet flow velocity conditions V_1_ and V_3_ at the T cycle (Figure 4a). Similarly, the maximum (18 mm/min) velocity was observed at rear region P1 and the outlet region for inlet flow velocity V_2_. The contour profile of axial velocity of the non-Newtonian viscosity formulation with physiological flow of sample S1 (model II) had maximum axial velocity on the rear region (P_1_ and P_2_) and neck region (P_1_ and P_2_) at the T/3 time cycle, as shown in Figure 4b. From Figure 4c, it can be observed that the contour plot of axial velocity for the Newtonian viscosity formulation with physiological flow of serpentine model S1 (model III) was similar to the axial velocity obtained for the non-Newtonian viscosity formulation with physiological flow of serpentine model 2 (model V). Such observation might be due to the serpentine channel orientation effect, irrespective of the hematocrit model of the working fluid flowing through the serpentine channels. The maximum velocity (20 mm/min) for both models was developed for the V_6_ condition on the periphery of point P_1_ of both serpentine models at T/3 s. For the non-Newtonian viscosity formulation with sinusoidal flow of serpentine model S2, the local maximum axial velocity (18 mm/min) was recorded on the periphery of the neck region (P_1_) and the periphery of the rear region (P_3_ at the T cycle, as shown in Figure 4d). Similarly, for the non-Newtonian viscosity formulation with physiological flow of serpentine model S2, it was observed that variations in axial velocity were measured at T/3 and T/2 s for inlet flow velocity conditions V_4_, V_5_, and V_6_. As depicted in Figure 4e, the maximum axial velocity was generated on the periphery of the neck region (P_1_), abdominal region (P_2_), and rear region (P_3_) at T/3 for inlet flow velocity conditions V_4_ and V_5_. From Figure 4b,e, it can be seen that the serpentine model S1 tended to have lower axial velocities than serpentine model S2 due to the larger radius of the curvature. Similarly, it was observed that the non-Newtonian viscosity produced a greater maximum axial velocity compared to the Newtonian viscosity formulation, as shown in Figure 4b,c.

**Figure 5:**
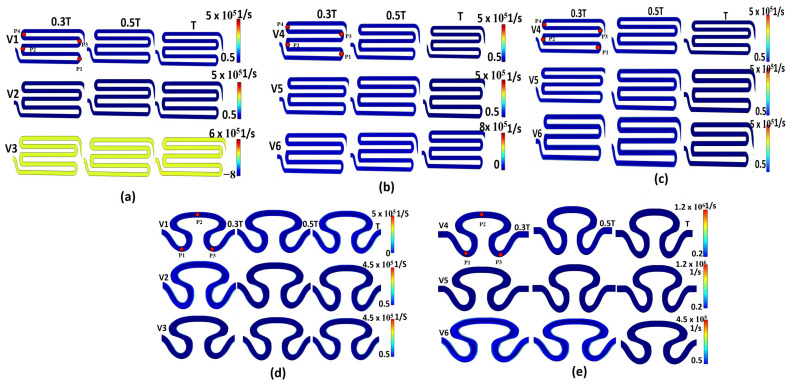
Contour plot of shear rate for different models: (**a**) model S1, non-Newtonian viscosity with sinusoidal flow; (**b**) model S1, non-Newtonian viscosity with physiological flow; (**c**) model S1, Newtonian viscosity with sinusoidal flow; (**d**) model S2, non-Newtonian viscosity with sinusoidal flow; (**e**) model S2, non-Newtonian viscosity with physiological flow.

The gradients in axial velocity were also evaluated in the form of shear rate. The variation of axial velocity for the Newtonian and non-Newtonian viscosity formulations induced a significant fluctuation in the shear rate. Evaluation of the shear rate close to the wall of the lumen is crucial for many physiological processes. Changes in the shear rate close to a wall can influence how well cells adhere to that surface. Figure 5a shows the contour plots of the shear rate for all models. The non-Newtonian viscosity formulation with sinusoidal flow of serpentine model S1 (model I, Table 2) resulted in a greater maximum shear rate (6 $\times \;10^{5}\;1/s$) for inlet flow velocity condition V_3_ compared to inlet flow conditions V_1_ and V_2_. This maximum (6 $\times \;10^{5}\;1/s$) shear rate was obtained in the rear region P_1_ for the T/3, T/2, and T cycles. A contour plot of shear rate of the non-Newtonian viscosity formulation with physiological flow of serpentine model S2 is shown in Figure 5b. Local minima and maxima shear rate magnitude were observed for inlet flow velocity condition V_6_ (0.5 $\times \;10^{5}\;1/s$ and 8 $\times \;10^{5}\;1/s$) near the wall of the rear region 1 of serpentine model S1 after one complete cycle. The contour plot of the Newtonian viscosity formulation with physiological flow of serpentine model S1 showed the maximum shear rate ($5\times 10^{5}\;1/s$) in the neck region 2 and rear region 1 at the T/3 and T/2 cycles for inlet flow boundary condition V_5_ (Figure 5c). For the non-Newtonian viscosity formulation with sinusoidal flow of serpentine model S2, the maximum shear rate ($5\times 10^{5}\;1/s$) was observed for inlet flow velocity condition V_1_ near the rear region (P_3_) at the T cycle, as shown in Figure 5d. The contour plot of shear rate for the non-Newtonian viscosity formulation of physiological flow of serpentine model S2 is shown in Figure 5e. It was observed that there was significant variation in the shear rate contour profile for inlet flow condition V_6_ in the periphery of the abdominal region (P_2_) at the T/2 cycle. From Figure 5b,c, it can be observed that the non-Newtonian formulation exhibited a greater maximum shear rate at the end of the T cycle compared to the Newtonian viscosity formulation. In comparison to serpentine model S2, the maximum shear rate was produced by serpentine model S1 due to the formation of eddies. 

**Figure 6:**
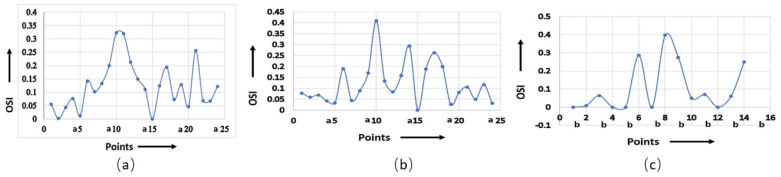
Calculated OSI for (**a**) model S1, Newtonian; (**b**) model S1, non-Newtonian; (**c**) model S2, non-Newtonian.

**Figure 7:**
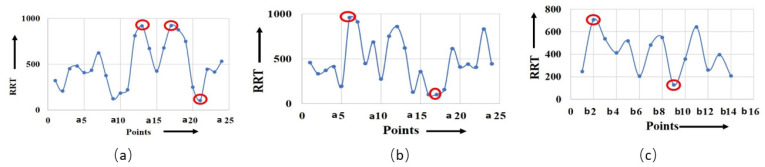
Calculated RRT for (**a**) model S1, Newtonian; (**b**) model S1, non-Newtonian; (**c**) model S2, non-Newtonian. The highlighted points show the maximum and minimum magnitude of RRT for respective models.

The shear rate is a crucial factor that influences the OSI value. As the shear rate increases, the material experiences higher deformation and viscous dissipation, resulting in higher flow asymmetry as well as OSI value. However, the relationship between the shear rate and OSI value is dependent on various material properties, i.e., viscoelasticity, atomic weight, concentration, and temperature of the working fluid. It was observed that the maximum OSI was obtained at point a10 for the non-Newtonian viscosity formulation of serpentine model S1, as shown in Figure 6a,b. Figure 6c shows the maximum OSI (0.4) at point b8 for the non-Newtonian viscosity formulation for serpentine model S2. The rate of change in OSI was maximum for point a10 for the non-Newtonian and Newtonian viscosity formulations in serpentine model S1, whereas the maximum rate of change in OSI was observed for point b8 for the non-Newtonian viscosity formulation in serpentine model S2. There was a positive correlation between OSI and RRT. The regions with low OSI tended to have longer RRTs, indicating that fluid elements reside longer in these regions prior to leaving. This is due to the fact that low OSI regions are typically associated with regions of flow separation and recirculation, where fluid is confined and complex flow patterns occur. Figure 7a demonstrates that the maximum (910) and minimum (100) magnitudes of RRT were measured for the Newtonian viscosity of serpentine model S1 at points a17 and a21, respectively. Similarly, for the non-Newtonian viscosity formulation of serpentine model S1, the maximum (950) and minimum (100) magnitudes of RRT were measured for points a6 and a17, respectively (Figure 7b). For serpentine model S2, the maximum (700) and minimum (110) magnitude of RRT were produced at b2 and b9 points, respectively, as shown in Figure 7c. From Figure 7b,c, it can be seen that the maximum RRT magnitude of serpentine model S1 was higher in comparison to serpentine model S2 due to surface tension, which caused particles to adhere to the wall of the channel. The RRT values for serpentine model S1 was higher in the regions for ¼ length of the channel from the inlet and outlet. In addition to that, the RRT curve of the non-Newtonian viscosity formulation of serpentine model S1 showed maximum negative gradient, while the serpentine model S2 had the maximum positive gradient. A big difference in RRT between the upper and lower walls was observed at points a11 and a12 for the Newtonian viscosity formulation in serpentine model S1. Similarly, the maximum RRT difference was observed at points a5 and a6 for a non-Newtonian viscosity formulation of serpentine model S1. Serpentine model S2 showed the maximum RRT difference at points b1 and b2 for non-Newtonian viscosity.

Deviations in hemodynamic parameters, including pressure, axial velocity, and shear rate, are presented in Table 3. The maximum and minimum deviation of pressure was reported for V_4_ and V_6_ inlet flow conditions at points P_1_ and P_2_, respectively, at the end of one complete flow cycle in the S2 model. Maximum deviation in axial velocity was observed for the V_6_ inlet flow condition at point P_2_ during the T/2 flow cycle in model II (as shown in Table 4). From the above observation, it was found that the maximum deviations of pressure (39.98%), axial velocity (25.8%), and shear rate (70.15%) were obtained for the non-Newtonian viscosity formulation with physiological flow for serpentine model S2 near point P_2_ at the T and T/2 cycles.

## 4. Discussion

Variations in working fluid rheology, orientation of serpentine geometry, and the flow architecture of the working fluid at different regions of the serpentine vascular channel significantly affected the distribution of pressure, axial velocity, and shear rate of the flowing fluid. Dean number was employed to develop a relationship between the flow rate and the fluctuating fluid pressure in a curved channel [43,44]. For models I and III, the maximum downstream pressure was developed at the end of one complete cycle due to fully developed flow [45,46]. In microchannels [47,48], the flow restriction parameter R depended on the channel length, which caused maximum pressure to be generated at the inlet region for the case of the non-Newtonian viscosity formulation with sinusoidal and physiological flows in serpentine models S1 and S2. For the non-Newtonian formulation with physiological flow of serpentine model 2, minimal variations in the magnitude of the flow parameters were found due to insignificant variation of the pressure gradient at different flow fluxes.

Variations in the pressure gradient influenced the axial velocity for different study configurations. In the case of transient analysis, the maximum variations in axial velocity distribution were observed at the end of the complete cycle in models II and V. Serpentine structure, along with the rheology of the media, could be the cause of such a phenomenon [49]. For the non-Newtonian formulation with physiological flow in serpentine model S2 (model V), axial velocity near the wall stopped [50]. It was observed that for most of the conditions, the velocity variation was maximum at the T/3 flow cycle because the curvilinear shape of the proposed serpentine structures reduced the kinetic energy of the flowing particles with the development of the flow. Due to specific orientation of the serpentine conduits, fluid particles obtained higher kinetic energy at the initial flow phase T/3, and the particles therefore had higher velocity in the presence of maximum magnitude of pressure gradient in both the Newtonian and non-Newtonian viscosity formulations with physiological flow in the serpentine model S2 [51,52]. 

Variations in the axial velocity of fluid influenced the shear rate distribution inside the channel. For the non-Newtonian viscosity model with sinusoidal flow in serpentine model S1, the parabolic profile of the velocity distribution promoted particles to have prolonged attachment on the walls, resulting in higher magnitude of localized shear rate [53]. For models II, III, IV, and V, a minimal variation in shear rate was found due to a nominal variation in axial velocity with respect to the luminal radius of the serpentine channel [54,55]. 

Pressure gradient profiles were found to be heterogeneous in serpentine models. It resulted in the generation of distinct localized discrete axial vorticity (as shown in Table 4). The development of localized vortices due to the serpentine model was responsible for maximum velocity deviation, leading to the development of radial pressure gradient [56,57]. Furthermore, elevated radial pressure gradient influenced the radial flow of the media, thereby enhancing deviation in the shear rate of adjacent regions. Different flow waveforms (sinusoidal and physiological) altered the distribution of fluid particles in dynamic flow. It resulted in generation of maximum shear rate variation [58,59]. Narrow conduits of flow channel in between two adjacent serpentine structures promoted the development of uniform shear rate [60] with minimal variation. Corners and bending structures complicated fluid flow structures through the microchannel. It resulted in recirculation or flow separation. These complex flow patterns caused variations in the residence time of the fluid [61,62]. Since, the power-law model was used to represent the non-Newtonian nature of the fluid flow, the flow rate was not linear in relation to the applied shear stress, thereby affecting the RRT distribution.

Higher values of OSI in the non-Newtonian fluid formulation was due to the presence of nonlinearity in the rheological behavior of the non-Newtonian viscosity fluid. At point a10 for the non-Newtonian viscosity formulation of serpentine model S2, the shear rate was higher, which enhanced the power-law index, causing an increase in the OSI magnitude. In the case of Newtonian fluid, the shear stress and shear rate were in phase, which restricted the fluctuation of OSI [14,63]. In serpentine model S1, the central axis had greater degree of curvature in comparison to serpentine model S2. Hence, the direction of the flow changed gradually. Minute changes in flow direction made it less likely for the flow profile to split and create eddies. Hence, it had smoother flow with lower OSI values. In addition to the serpentine model S2, the flow path was also divided into multiple channels. The flow then recombined in the downstream region. Formation of multiple channels disturbed the flow patterns [64] and flow separation, leading to increased flow variation and elevated OSI values. The fluid’s surface tension was responsible for the creation of menisci at the corners of serpentine model S1, resulting in a change in the flow pattern and velocity of the fluid, which increased the RRT value [65,66].

## 5. Conclusions

Hemodynamic properties of biofluid, such as pressure, axial velocity, and shear rate, were found to play an essential role in inducing physiological behavior and promoting cell adhesion properties under in vitro conditions on the inner lining of a serpentine plane. Fluid in the serpentine channel generated longitudinal stress, which in turn influenced cellular functionality. This numerical analysis evaluated the impact of Newtonian and non-Newtonian viscosity models on various fluid dynamic parameters of the culture media–serpentine wall interaction. Simultaneously, the local maxima and minima of different hemodynamic parameters were identified at specified regions of the serpentine models (model S1: points P_1_ to P_4_; model S2: points P_1_ to P_3_) as potential slots of effective cellular proliferative zones over the inner lining of the serpentine channel. It was observed that the non-Newtonian viscosity formulation possessed maximum variation in rheological properties compared to the Newtonian viscosity model. In comparison to serpentine model S2, the formation of eddies within serpentine model S1 was more frequent. It produced maximum variation in pressure, axial velocity, and shear rate. OSI and RRT distribution mapping also provided suitable marked regions in serpentine model S1 and S2, which could support maximum growth of cells.

## Figures and Tables

**Table 1:** Inlet flow velocity function for V_1_ to V_6_.

Inlet Velocity	Model	Parameters Value	Function
Sinusoidal flow (SF)	V_1_	f = 1.25 Hz, t = 0 to 1 s	V_1_(t) = 4.48× $\sin\left[2\times \pi \times f\times t\right]$
	V_2_		V_2_(t) = 4.62× $\sin\left[2\times \pi \times f\times t\right]$
	V_3_		V_3_(t) = 4.76× $\sin\left[2\times \pi \times f\times t\right]$
Physiological flow (PF) 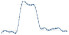	V_4_	t = 0 to 1 s, a_1_ = 3.04, b_1_ = 0.8634, c_1_ = −0.07247, a_2_ = 1.24, b_2_ = 14.02, c_2_ = 2.164, a_3_ = 2.619, b_3_ = 5.81, c_3_ = −0.5393, a_4_ = 0.4497, b_4_ = 24.25, c_4_ = 1.474, a_5_ = 0.3847, b_5_ = 32.5, c_5_ = −2.181, a_6_ = 0.4739, b_6_ = 20.77, c_6_ = 0.4904, a_7_ = 0.2708, b_7_ = 37.65, c_7_ = 2.495, a_8_ = 0.1386, b_8_ = 52.47, c_8_ = −0.7126	V_4_ (t) = $a_{1}\times sin[b_{1}\times t]+\;c_{1}]+a_{2}\times sin[b_{2}\times t]+c_{2}]+\;a_{3}\times sin[b_{3}\times t]+c_{3}]+a_{4}\times \;sin[b_{4}\times t]+c_{4}]+a_{5}\times \;sin[b_{5}\times t]+c_{5}]+a_{6}\times \;sin[b_{6}\times t]+c_{6}]+a_{7}\times \;sin[b_{7}\times t]+c_{7}]+a_{8}\times \;sin[b_{8}\times t]+c_{8}]$
	V_5_	t = 0 to 1 s, a_1_ = 2.721, b_1_ = 1.588, c_1_ = −0.4734, a_2_ = 1.364, b_2_ = 13.48, c_2_ = 2.332, a_3_ = 3.081, b_3_ = 5.052, c_3_ = −0.1051, a_4_ = 0.3837, b_4_ = 24.37, c_4_ = 1.263, a_5_ = 0.4042, b_5_ = 32.44, c_5_ = −2.133, a_6_ = 0.4619, b_6_ = 19.567, c_6_ = 0.9404, a_7_ = 0.2744, b_7_ = 37.67, c_7_ = 2.482, a_8_ = 0.1435, b_8_ = 52.48, c_8_ = −0.7228	V_5_ (t) = $a_{1}\times sin[b_{1}\times t]+\;c_{1}]+a_{2}\times sin[b_{2}\times t]+c_{2}]+\;a_{3}\times sin[b_{3}\times t]+c_{3}]+a_{4}\times \;sin[b_{4}\times t]+c_{4}]+a_{5}\times \;sin[b_{5}\times t]+c_{5}]+a_{6}\times \;sin[b_{6}\times t]+c_{6}]+a_{7}\times \;sin[b_{7}\times t]+c_{7}]+a_{8}\times \;sin[b_{8}\times t]+c_{8}]$
	V_6_	t = 0 to 1 s, a_1_ = 2.864, b_1_ = 1.405, c_1_ = −0.3165, a_2_ = 1.322, b_2_ = 13.96, c_2_ = 2.122, a_3_ = 3.008, b_3_ = 5.506, c_3_ = −0.3209, a_4_ = 0.7938, b_4_ = 22.63, c_4_ = 2.259, a_5_ = 0.4209, b_5_ = 31.95, c_5_ = −1.982, a_6_ = 0.8909, b_6_ = 20.65, c_6_ = 0.3462, a_7_ = 0.3141, b_7_ = 37.4, c_7_ = 2.577, a_8_ = 0.1474, b_8_ = 52.51, c_8_ = −0.7326	V_6_ (t) = $a_{1}\times sin[b_{1}\times t]+\;c_{1}]+a_{2}\times sin[b_{2}\times t]+c_{2}]+\;a_{3}\times sin[b_{3}\times t]+c_{3}]+a_{4}\times \;sin[b_{4}\times t]+c_{4}]+a_{5}\times \;sin[b_{5}\times t]+c_{5}]+a_{6}\times \;sin[b_{6}\times t]+c_{6}]+a_{7}\times \;sin[b_{7}\times t]+c_{7}]+a_{8}\times \;sin[b_{8}\times t]+c_{8}]$

**Table 2:** Different boundary conditions for the numerical study.

Model	Serpentine Model	Viscosity Formation	Inlet Boundary Condition	Inlet Velocity Conditions
I	S1	Non-Newtonian (NN)	Sinusoidal flow (SF)	V_1_
				V_2_
				V_3_
II		Non-Newtonian (NN)	Physiological flow (PF)	V_4_
				V_5_
				V_6_
III		Newtonian (N)	Physiological flow (PF)	V_4_
				V_5_
				V_6_
IV	S2	Non-Newtonian (NN)	Sinusoidal flow (SF)	V_1_
				V_2_
				V_3_
V		Non-Newtonian (NN)	Physiological flow (PF)	V_4_
				V_5_
				V_6_

**Table 3:** Order of accuracy and grid convergence index for the pressure variable.

Variable	Model and Point	r	*p*	Fs	${GCI}_{23}$ (%)	${GCI}_{12}$ (%)
Pressure	S1, P_1_	1.793	0.794	1.25	4.4	2.7
	S2, P_1_	1.795	0.623		3.2	4

**Table 4:** Calculated deviation for pressure, shear rate, and velocity.

S.No.	Models	Parameter	Point	Time	Deviation	
1	S1_NN_NS_SF_V1, V2, V3	Pressure	P_1_ (V_3_) percent [V_2_, V_3_]	0.3	Minimum	2.57
			P_3_ (V_3_) percent [V_3_, V_1_]	1	Maximum	37.86
		Shear rate	P_4_ (V_1_) percent [V_1_, V_2_]	0.5	Minimum	0.26
			P_4_ (V_2_) percent [V_2_, V_3_]	1	Maximum	67.36
		Velocity	P_4_ (V_2_) percent [V_1_, V_2_]	0.5	Minimum	2.33
			P_3_ (V_3_) percent [V_3_, V_1_]	0.3	Maximum	10.18
2	S1_NN_NS_PF_V4, V5, V6	Pressure	P_1_ (V_5_) percent [V_4_, V_5_]	1	Minimum	1.94
			P_3_ (V_6_) percent [V_4_, V_6_]	0.3	Maximum	37.46
		Velocity	P_1_ (V_5_) percent [V_4_, V_5_]	0.5	Minimum	2.77
			P_3_ (V_6_) percent [V_4_, V_6_]	0.3	Maximum	7.65
3	S1_N_NS_PF_V4, V5, V6	Pressure	P_2_ (V_6_) percent [V_4_, V_5_]	1	Minimum	3.02
			P_3_ (V_6_) percent [V_6_, V_4_]	0.3	Maximum	37.46
		Velocity	P_1_ (V_6_) percent [V_5_, V_6_]	1	Minimum	2.64
			P_3_ (V_6_) percent [V_6_, V_5_]	1	Maximum	15.15
4	S2_NN_NS_SF_V1, V2, V3	Pressure	p_1_ (V_3_) percent [V_2_, V_3_]	0.5	Minimum	3.33
			p_1_ (V_1_) percent [V_3_, V_1_]	1	Maximum	10.42
		Shear rate	p_1_ (V_2_) percent [V_2_, V_1_]	1	Minimum	2.10
			p_2_ (V_1_) percent [V_1_, V_3_]	0.3	Maximum	13.55
		Velocity	p_3_ (V_2_) percent [V_3_, V_1_]	0.5	Minimum	2.91
			p_3_ (V_3_) percent [V_1_, V_3_]	1	Maximum	5.86
5	S2_NN_NS_PF_V4, V5, V6	Pressure	P_2_(V_6_) percent [V_5_, V_6_]	1	Minimum	0.009
			P_1_ (V_4_) percent [V_6_, V_4_]	1	Maximum	39.98
		Shear rate	P_1_(V_5_) percent [V_5_, V_6_]	1	Minimum	0.28
			P_2_ (V_6_) percent [V_5_, V_6_]	0.5	Maximum	70.15
		Velocity	P_3_ (V_6_) percent [V_5_, V_6_]	0.5	Minimum	0.140
			P_2_(V_6_) percent [V_5_, V_6_]	0.5	Maximum	25.8
